# Electroencephalographic study of the Error Related Negativity in patients suffering from treatment-resistant obsessive-compulsive disorder

**DOI:** 10.1192/j.eurpsy.2023.526

**Published:** 2023-07-19

**Authors:** I. Wassouf, J. Dampuré, D. Doolub, G. Harika-Germaneau, N. Jaafari, N. Vibert

**Affiliations:** ^1^Centre de Recherches sur la Cognition et l’Apprentissage (CeRCA), CNRS, Université de Poitiers, Université de Tours, Poitiers; ^2^Centre Hospitalier Nord Deux-Sèvres, Thouars; ^3^Unité de Recherche Clinique Pierre Deniker, Centre Hospitalier Henri Laborit, Poitiers; ^4^Département de Psychologie, Université Catholique de l’Ouest, Niort; ^5^Faculté de Médecine, Université de Poitiers, Poitiers, France

## Abstract

**Introduction:**

The physiopathology of patients with treatment-resistant obsessive-compulsive disorder (OCD) may differ from that of treatment-responsive patients. Cognitive evoked potentials may be one of the ways to detect these differences. The error-related negativity (ERN) is an electrophysiological correlate of error detection during the execution of a motor task, which is larger in patients with OCD than in typical people. According to the literature, the ERN could vary according the patients’ treatment-resistance.

**Objectives:**

The main goal of this study was to assess whether the ERN, which begins 20 ms before the motor response and reaches its maximum 80 ms later, was different between non-resistant and highly resistant OCD patients.

**Methods:**

Forty-seven OCD patients and their age- and gender-matched controls were asked to perform a flanker task while the potentials evoked by their motor responses were recorded. For each participant, the difficulty of the task was adjusted to get an error rate of about 20%. Treatment-resistance was evaluated using Pallanti and Quercioli’s (Prog Neuropsychopharmacol Biol Psychiatry 2006; 30 400-412) resistance scales.

**Results:**

In all participants, response errors evoked an ERN at fronto-central electrodes [Fz, FCz, red and green lines on the figure], whereas the negativity was absent or smaller for correct responses (black and blue lines on the figure, both *p*s < .01). As expected, the ERN of OCD patients was consistently larger and of longer duration than that of control participants (compare the green and blue lines with the red and black ones, respectively). Interestingly, the amplitude of the potentials evoked by correct responses at central and centro-parietal electrodes on the left side of the brain [C5, C3, CP5] was significantly less negative in treatment-resistant patients (all *p*s < .05). In control participants, the ERN recorded at fronto-central electrodes were followed by a positive wave which reached its maximum between 170 and 270 ms after the response, and was larger after errors than after correct response. In OCD patients in contrast, this positive wave was absent whether the response was correct or not.

**Image:**

**Figure:**
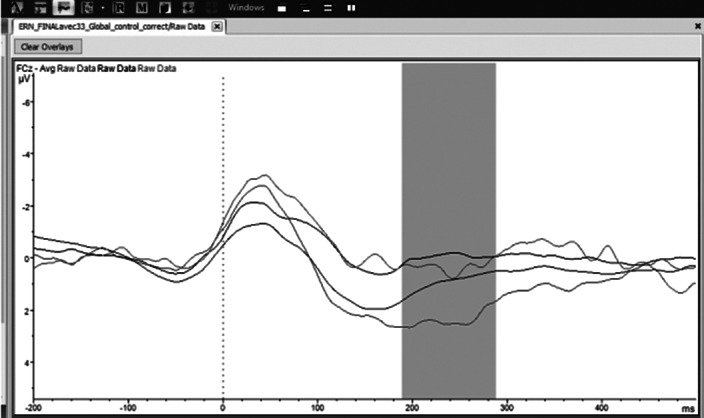

**Conclusions:**

The significant correlation observed between OCD patients’ treatment resistance and the potential evoked by correct responses on the left side of the brain suggest that this potential could be used as a marker of treatment-resistance. The absence in OCD patients of the positive wave that follows the ERN in control participants suggests that OCD patients were not fully aware of whether or not their response was correct.

**Disclosure of Interest:**

None Declared

